# Multiple Correspondence Analysis and HLA-Associations of Organ Involvement in a Large Cohort of African-American and European-American Patients with Sarcoidosis

**DOI:** 10.1007/s00408-023-00626-6

**Published:** 2023-06-15

**Authors:** Astrid Rasmussen, Bryan A. Dawkins, Chuang Li, Nathan Pezant, Albert M. Levin, Benjamin A. Rybicki, Michael C. Iannuzzi, Courtney G. Montgomery

**Affiliations:** 1 Genes and Human Disease Program, Oklahoma Medical Research Foundation, 825 NE 13th, Research Tower, Suite 2202, Oklahoma City, Ok 73104 USA; 2 Department of Public Health Sciences, Henry Ford Health System, Detroit, MI USA; 3 Department of Medical Education, City University of New York School of Medicine, New York, NY USA

**Keywords:** Sarcoidosis, Precision Medicine, Multiple Correspondence Analysis, African-American ancestry

## Abstract

Sarcoidosis is a systemic granulomatous disease with predominant pulmonary involvement and vast heterogeneity of clinical manifestations and disease outcomes. African American (AA) patients suffer greater morbidity and mortality. Using Multiple Correspondence Analysis, we identified seven clusters of organ involvement in European American (EA; n = 385) patients which were similar to those previously described in a Pan-European (GenPhenReSa) and a Spanish cohort (SARCOGEAS). In contrast, AA (n = 987) had six, less well-defined and overlapping clusters with little similarity to the cluster identified in the EA cohort evaluated at the same U.S. institutions. Association of cluster membership with two-digit HLA-DRB1 alleles demonstrated ancestry-specific patterns of association and replicated known HLA effects.

These results further support the notion that genetically influenced immune risk profiles, which differ based on ancestry, play a role in phenotypic heterogeneity. Dissecting such risk profiles will move us closer to personalized medicine for this complex disease.

**Supplementary Information:**

The online version contains supplementary material available at 10.1007/s00408-023-00626-6.

## Introduction

The pathologic hallmark of sarcoidosis is granulomatous inflammation primarily of the lungs and intrathoracic lymph nodes but which can affect a wide variety of organs. A puzzling aspect of the disease is the heterogeneity that characterizes it. Many patients eventually clinically resolve, sometimes spontaneously and without treatment. However, up to one-third of the cases will have a protracted course with significant morbidity and health care utilization, and ~ 10% will progress to irreversible fibrotic disease [1]. The number and pattern of organs involved can vary greatly and some subphenotypes impact quality of life more than others (i.e., neurosarcoidosis and cardiac sarcoidosis have greater mortality than cutaneous sarcoidosis) [2, 3]. It is unclear what determines this variability, but there is epidemiologic evidence that a combination of genetic variants and the influence of environmental factors contribute to phenotypic variability. Socioeconomic factors and access to care have also been associated with divergent outcomes [4–7].

Attempts have been made at identifying predictors of worse prognosis, poor quality of life, and increased need for aggressive treatment [6]. Schupp et al. performed multidimensional correspondence analysis (MCA) and clustering of a cohort of > 2000 Caucasian sarcoidosis patients and identified five distinct subgroups based on patterns of organ involvement. The SARCOGEAS study group, analyzing data from 1230 Spanish patients replicated these organ associations [8]. However, the patterns of organ involvement have not been analyzed in populations of non-European ancestry. Our aim was to assess a large cohort of African-Americans (AA) and European-Americans (EA) recruited in the United States to characterize the patterns of organ clustering, compare them with the reported European findings, and assess association with HLA-DRB1, the most confirmed genetic association with sarcoidosis.

## Methods

### Participants

This study was conducted with data from 2072 sarcoidosis cases (1539 AA and 571 EA) originally recruited into three past cohorts, specifically “A Case Control Etiologic Study of Sarcoidosis (ACCESS) Group”[9], “Sarcoidosis Genetic Analysis (SAGA) study”[10], and “Henry Ford Family Study (HFFS)” [11]. (Table 1). Patients in these cohorts were primarily ascertained in outpatient visits to pulmonary clinics and organ involvement was assessed at a single timepoint.

**Table 1:** African American and European American Sarcoidosis Cohorts

	African American			European American		
	Complete Cohort n = 1593	Clustered Subset n = 987		Complete cohort n = 517	Clustered Subset n = 385	
Sex: female	1128 (73.3)	716 (72.5)		301 (58.2)	221 (57.4)	
Age at diagnosis (mean ± SD)	48.5 ± 8.1	48.2 ± 7.6		n/a	n/a	

Organ Involvement (ACCESS or WASOG)^1^	Organ involved n (%)	Organ involved n (%)	Missing data n (%)	Organ involved n(%)	Organ involved n(%)	Missing data n(%)
Pulmonary	1260 (98)	965 (97.8)	22 (2.2)	n/a	369 (95.8)	16 (4.2)
Neurological	104 (10.5)	101 (10.2)	12 (1.2)	n/a	34 (8.8)	0 (0)
Ocular	288 (28.9)	274 (27.8)	13 (1.3)	n/a	43 (11.2)	0 (0)
Cardiac	40 (4.0)	40 (4.1)	4 (0.4)	n/a	11 (2.9)	0 (0)
Liver	207 (20.7)	197 (20.0)	5 (0.5)	n/a	24 (6.2)	0 (0)
Spleen	82 (8.2)	79 (8.0)	6 (0.6)	n/a	28 (7.3)	0 (0)
Calcium/Vitamin D	59 (6.7)	57 (5.8)	123 (12.5)	n/a	31 (8.1)	0 (0)
Renal	21 (2.1)	20 (2.0)	3 (0.3)	n/a	5 (1.3)	0 (0)
Skin	428 (42.1)	401 (40.6)	4 (0.4)	n/a	84 (21.8)	0 (0)
Salivary Glands	78 (8.8)	72 (7.3)	115 (11.7)	n/a	28 (7.3)	0 (0)
Muscle	32 (3.6)	32 (3.2)	113 (11.4)	n/a	13 (3.4)	0 (0)
Bone Marrow	94 (9.4)	92 (9.3)	4 (0.4)	n/a	5 (1.3)	0 (0)
Extra-Thoracic LN	214 (21.3)	202 (20.5)	6 (0.6)	n/a	49 (12.7)	0 (0)
Upper Respiratory Tract	97 (16.8)	90 (9.1)	428 (43.4)	n/a	0 (0.0)	385 (100)
Bone and Joint	96 (12.1)	92 (9.3)	201 (20.4)	n/a	85 (22.1)	0 (0)

Comparison of each complete sub−cohort by race to the subset with enough data to be clustered

^1^Based on the ACCESS or WASOG Organ Involvement Instruments [9, 12]

^2^For the subsets of patients included in the clustering analysis, the number and percentage of patients with each organ involved is shown followed by the number of subjects (%) for whom the corresponding data are missing

n/a: Data not available

Organ involvement was assessed based on either the ACCESS or the WASOG Organ Assessment Tools [9, 12]; only subjects with confirmed sarcoidosis and data documenting a minimum of two organs affected were considered useful for analysis. Furthermore, adjudication of involvement of at least 50% of the organs of the WASOG Assessment Tool was required for a subject to be included. Thus, the final dataset consisted of 987 AA and 385 EA cases. Two-digit DRB1 HLA genotype data for the complete cohort were available from previous studies [13].

### Clustering

Multiple correspondence analysis (MCA) of the organ involvement, an analog to principal components analysis (PCA) for categorical data, was used for dimension reduction prior to clustering using the FactoMineR package in [14]. Hierarchical clustering with Ward’s linkage method on the first 5 MCA dimensions was used to derive clusters. Cluster stability was assessed using bootstrap resampling (n = 1000 samples), which was implemented with the fpc package in R [15]. The total number of clusters was chosen using the average Jaccard similarity metric across bootstrap samples in order to maximize the total number of stable clusters, defined heuristically as those with bootstrap average Jaccard similarity exceeding 0.7.

### Statistical Comparisons

A hypergeometric test was used for assessing the pairwise association between organs and clusters, where the association was considered statistically significant if its hypergeometric p-value was less than 0.05. An organ was considered “enriched” within a cluster if its within-cluster frequency exceeded its background frequency; “depletion” was defined similarly, only the relationship between the organ’s within-cluster frequency and its background frequency was reversed. The standard normal quantile corresponding to the hypergeometric p-value, scaled positively (enrichment) or negatively (depletion) was used to visualize the association between organs and clusters. Association of MCA cluster membership with HLA-DRB1 alleles (additive model) was determined using logistic regression with inclusion in a given cluster versus not as the outcome variable. The HLA allele was the independent variable, with sex and the first 4 principal components for ancestry as covariates. Statistical significance was defined as p < 0.05.

## Results

**Fig. 1:**
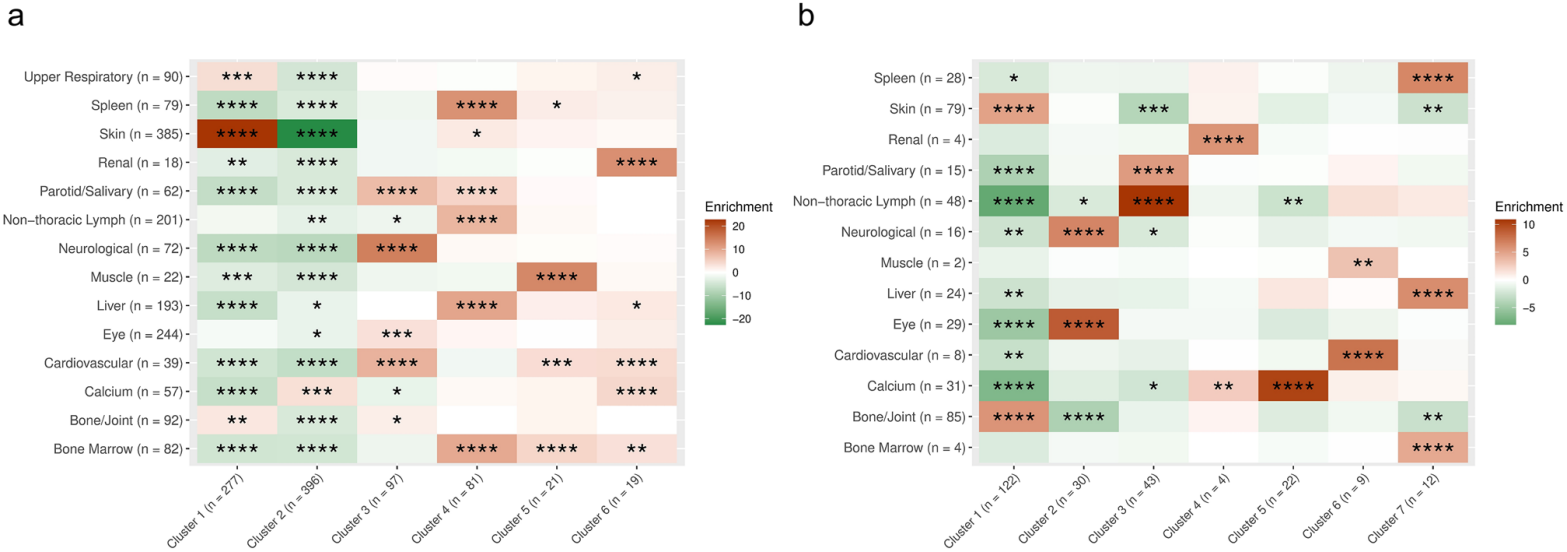
Organ enrichment by cluster in African Americans and European Americans. Panel **a** shows the patterns of organ enrichment in African Americans and Panel **b** shows the patterns of organ enrichment in European Americans. Rows correspond to the involvement of each organ; columns correspond to clusters. The color scale is derived from standard normal quantiles corresponding to hypergeometric p-values for the pairwise association between organ and cluster. The relationship between an organ’s within-cluster frequency and its background frequency determines the sign or direction of each quantile; positive quantiles denote greater within-cluster frequency (enriched, shown in shades of brown) and negative quantiles denote lesser within-cluster frequency (depleted, shown in shades of green). Asterisks represent hypergeometric p-value magnitude (**** $P<0.0001$, ***$P<0.001$, **$P<0.01$, *$P<0.05$)

Subjects included in the clustering analyses were a subset of the complete cohorts but are demographically comparable to the subjects excluded due to missing data (Table 1). The clusters identified are named by the primary organ involved (Fig. 1a, b).

### African-American Sarcoidosis Cases

AA patients were predominantly female (~ 73%) with a mean age at diagnosis of 48.5 years (SD 8.1; median 49). The most frequently affected organs were the lungs (98%) followed by skin (41%), eyes (28%), and extrathoracic lymph nodes (21%).

**Fig. 2:**
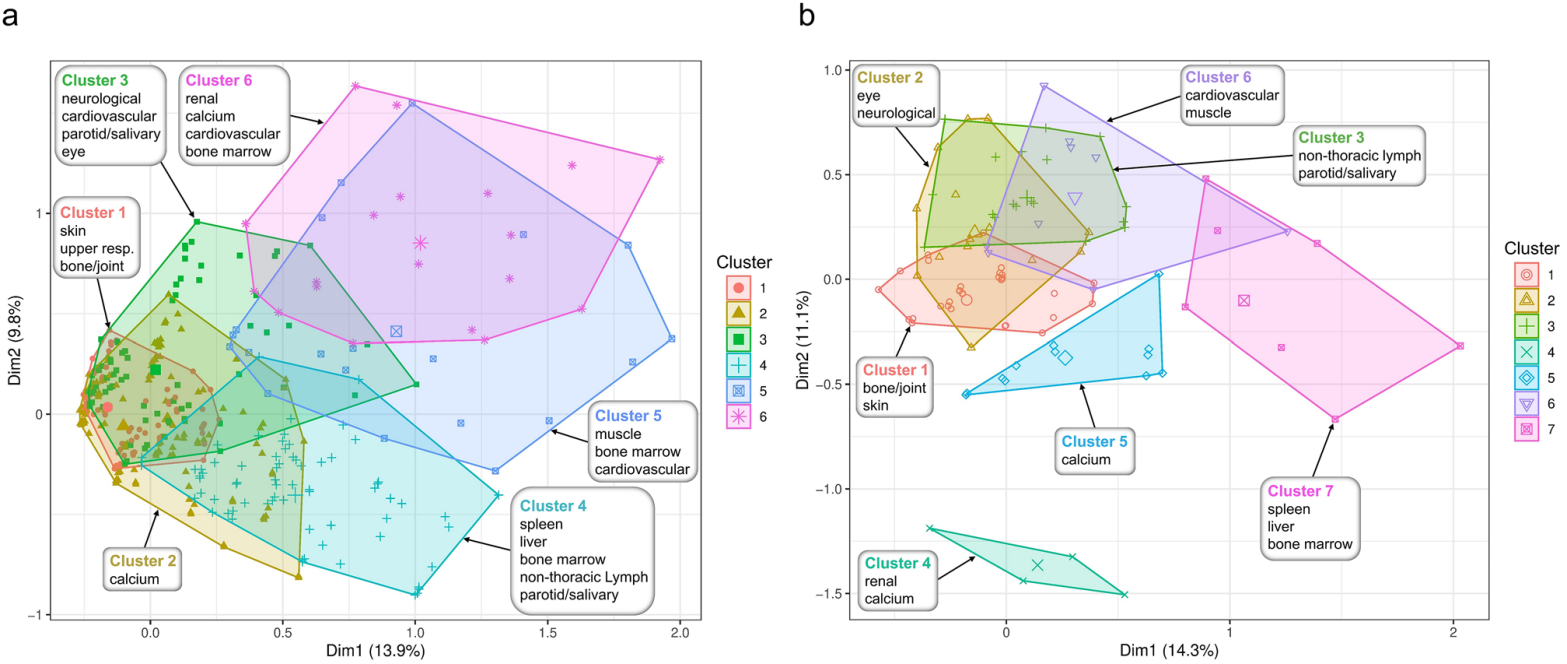
Clustering of systemic sarcoidosis phenotypes in African Americans and European Americans. Each point represents a sarcoidosis-affected individual’s contribution to the first two principal dimensions (Dim1 and Dim2), calculated with Multiple Correspondence Analysis (MCA) on organ involvement data. Enriched (or over-represented) affected organs are used for annotating each cluster. Panel **a** shows the clustering of organ involvement in African-Americans and Panel **b** shows the clustering of organ involvement in European-Americans

Six distinct multi-organ clusters were identified in AA (Fig. 1a), excluding a pulmonary-only subset of subjects not included in MCA (n = 96). The largest cluster was the calcium cluster (n = 396) followed by the skin cluster (n = 277). Intermediate in frequency were the neuro/ocular/cardiac/salivary gland and the abdominal organs/extrathoracic lymph nodes clusters (n = 97 and 81, respectively). Two additional small clusters, with a moderate overlap of the organs involved, were the muscle/cardiac/bone marrow (n = 21) and renal/cardiac/calcium (n = 19) clusters (Fig. 2a). The proportional contribution of each organ to the cluster is shown in Fig. 2a. Age and sex did not differ between the clusters.

Statistically significant association with common HLA-DRB1 alleles was found for all of the clusters except the calcium/vitamin D cluster (2) (Supplemental Table 1). Interestingly, the association of the cardiovascular/muscle/calcium cluster (6) to DRB1*01:02 is consistent with a previous investigation of the larger cohort from which this sample was derived showing association of this allele with persistent disease in AA [16].

### European-American Sarcoidosis Cases

Females were the majority of EA cases (~ 58%) and EA mainly presented with pulmonary (95.8%), cutaneous (21.8%), bone/joint (22.1%), and extrathoracic lymph node (21.8%) involvement. Seven multi-organ clusters, excluding a pulmonary-only subset of subjects not included in MCA (n = 143), were identified (Fig. 1b). These are different from those affecting AA subjects and resemble previous descriptions of organ involvement patterns in European populations [8, 17].

The largest cluster identified was bone/joint/skin (n = 122), similar to the musculoskeletal-cutaneous [17] and musculoskeletal and skin clusters [8] previously reported. The extrathoracic lymph nodes/salivary glands cluster (n = 43), partially replicates the adenopathic anatomic cluster [8], while the neurological/ocular (n = 30) cluster mimics the ocular-cardiac-cutaneous-central nervous system [17] and the anatomic neuro-ocular clusters [8]. A fourth cluster was calcium (n = 22) with no direct equivalent in previous studies. Smaller clusters were spleen/liver/bone marrow, similar to Schupp et al.’s abdominal organs cluster [17] and the anatomic hepatosplenic cluster [8]. Very small clusters (heart-muscle and kidney/calcium; Fig. 2b) would fit within the extrapulmonary involvement cluster [17].

All clusters except the calcium cluster had significant association with HLA-DRB1 alleles (Supplemental Table 1). Notably, we observed association of DRB1*03:01, which has previously been associated with resolving disease [18], with involvement of organs typically associated with disease resolution such as the bone/joint/skin cluster (OR = 2.79; 95% CI 1.73–4.55; p = 2.92E-05) while being protective for inclusion in the extrathoracic lymph node cluster (OR = 0.27; 95% CI 0.06–0.73; p = 0.028). Additional relevant associations were with various DRB1*04 alleles. We observed association of *04:01 with the neurological/ocular cluster (cluster 2; OR = 6.48; 95% CI 2.72—15.32; p = 1.97E-05); this allele has previously been associated with ocular involvement [19, 20]. DRB1*04 has also been associated with hypercalcemia [21] and we show association of the calcium/renal cluster with DRB1*04:05 (OR = 36.75; 95% CI 1.39–618.85; p = 0.011).

## Discussion

Sarcoidosis can be a benign disease but for many patients, it results in a debilitating condition that negatively impacts their quantity and quality of life. Understanding the factors that influence the patterns of organ involvement and overall heterogeneity of the disease is important for opportune diagnosis, early management of potentially serious complications, and future targeted therapies. We and others have analyzed the aggregation of disease manifestations and identified patterns occurring in specific populations. We present novel disease clusters for American sarcoidosis patients of African descent. Unfortunately, the AA organ clusters were less defined and overlapped significantly in the first two principal dimensions; this complexity required analyses in 5 dimensions to attain clear separation.

In contrast, the EA clusters were tighter and while they partially overlapped, three studies have replicated similar patterns supporting their value in the appropriate populations [8, 17].

We acknowledge the limitations of our study which include, more prominently for the EA cohort, a large proportion of missing data, and the use of mathematical modeling for clinical medicine. The goal of methods such as MCA and hierarchical clustering, is to reduce the noise present in data with a large number of variables while retaining the signal of interest and clarifying underlying patterns. As such, they serve to outline steps towards testing hypotheses and validating relationships by other means. [22, 23]

While genome-wide genotyping data and admixture proportions are available for the patients in the cohort described herein, we did not find any correlation between levels of admixture (i.e., the relative contribution of African and non-African ancestry) and specific phenotypic clusters. Each organ cluster is small and thus underpowered to conduct a genome-wide association scan, but additional candidate gene studies, similar to what we present for loci within HLA could give insight into ancestry-specific genetic influences on phenotypic heterogeneity. The overall non-African ancestry in this AA cohort is ~ 17% which is slightly less than other reports of black Americans [24], and thus, our findings may not be applicable to black populations with different origins or admixture patterns.

Nevertheless, the clear phenotypic differences we observed between the AA and EA subcohorts and our observation of HLA-DRB1 associations to specific patterns of organ involvement support ancestry-specific differences likely resulting from distinct social, cultural, and environmental factors in the setting of unique genetic determinants. Future research should be centered around the multi-omic deconstruction of sarcoidosis in people of all racial backgrounds.

## Supplementary Information

Below is the link to the electronic supplementary material.

Supplementary file1 (PDF 20 KB)
