# Supplementary material for: Multiple Correspondence Analysis and HLA-Associations of Organ Involvement in a Large Cohort of African-American and European-American Patients with Sarcoidosis
Source: Lung. 2023 Jun 15;201(3):297–302. doi: 10.1007/s00408-023-00626-6 (PMC10284928; doi:10.1007/s00408-023-00626-6)
Supplement: Supplementary file 1 — Supplementary file1 (PDF 20 KB) [file 408_2023_626_MOESM1_ESM.pdf]

Supplemental Table 1 - Association of organ clusters by race to HLA-DRB1 risk alleles

	Cluster	OR (95% CI)	p value	References
**African American**				
DRB1*01:02	Cardiovascular/Muscular/ Calcium	4.49 (1.5; 12.13)	0.004	[17]
DRB1*04:04	Abdominal organs/Extrathoracic	6.74 (1.72; 23.2)	0.003	
DRB1*07:01	Skin	0.50 (0.30; 0.82)	0.008	
DRB1*08:04	Neuro/Ocular/Cardiac/ Salivary gland	2.38 (1.33; 4.11)	0.002	
DRB1*11:02	Muscle/Cardiac/Bone marrow	4.38 (0.98; 14.08)	0.024	
DRB1*12:02	Renal/Cardiac/Vitamin D	11.75 (0.57; 88.23)	0.035	
DRB1*15:01	Abdominal organs/Extrathoracic	2.40 (0.93; 5.44)	0.049	
**European American**				
DRB1*03:01	Bone/Joint/Skin	2.79 (1.73; 4.55)	2.92E-05	
DRB1*03:01	Extrathoracic lymph nodes/Salivary glands	0.27 (0.06; 0.73)	0.027	[19]
DRB1*04:01	Neurological/Ocular	6.48 (2.72; 15.32)	1.97E-05	[20, 21]
DRB1*04:05	Renal/Calcium	36.75 (1.39; 618.85)	0.011	[22]

Statistical significance was defined as $p<0.05$. Strict Bonferroni correction for multiple testing would result in significance at $p<0.002$, however, given that these HLA alleles are not independent, we chose the more liberal cutoff value of $p<0.05$.
